# EQ-5D-3L and EQ-5D-5L population norms for Thailand

**DOI:** 10.1186/s12889-024-18391-3

**Published:** 2024-04-22

**Authors:** Krittaphas Kangwanrattanakul, Christian U. Krägeloh

**Affiliations:** 1 Division of Social and Administrative Pharmacy, Faculty of Pharmaceutical Sciences, Burapha University, 169 Long-Hard Bangsaen Rd.,, Chonburi Mueang, 20131 Thailand; 2 Department of Psychology and Neuroscience, Auckland University of Technology, Auckland, New Zealand

**Keywords:** EQ-5D, Health related quality of life, Thai population, Population norms

## Abstract

**Background:**

The previous Thai norm-based scores for the EQ-5D-5L were established with Thai general population samples aged 20–70 years in 2019. Nevertheless, these values need to be updated after the COVID-19 pandemic because of its effects on both physical and mental health. This study therefore aimed to establish population norms of the Thai EQ-5D-3L, EQ-5D-5L and EQ-VAS scores as well as to estimate disutility values associated with self-reported main diseases.

**Methods:**

Individual face-to-face interviews were conducted with 2000 adult (age ≥ 18 years) members of the general Thai population to estimate norm-based scores. Each participant completed the EQ-5D-3L and EQ-5D-5L as well as questions related to their sociodemographic factors and self-reported main diseases. Responses to the two instruments were converted to health utility (HU) scores on the basis of available value sets. Descriptive statistics were used to report the norm-based scores stratified by age and sex categories. Response redistribution determining the response consistency between EQ-5D versions was investigated. The HU score agreement from those two instruments was investigated using intraclass correlation coefficient (ICC). Tobit regression models were employed to investigate the relationships between sociodemographic factors and HU and EQ-VAS scores. Moreover, it was used to estimate the disutility values associated with self-reported main diseases.

**Results:**

The means (percentage of ceiling effects) of EQ-5D-3L, EQ-5D-5L, and EQ-VAS scores were 0.845 (57.80%), 0.923 (49.05%), and 79.83 (3.20%), respectively. The average percentage of inconsistent response was 1.09%. A good agreement level was found between both EQ-5D versions with the ICCs of 0.789 (95% CI: 0.558–0.878). Female, older, and unemployed participants and those with BMI ≥ 30 reported lower EQ-5D-3L and EQ-5D-5L than their counterparts. Bone/Joint disorder and stroke contributed to the largest disutility value for those two instruments.

**Conclusions:**

The Thai norm-based scores from those two instruments were diminished when advancing age and among female, unemployed, and obese (BMI ≥ 30) participants. It is expected to provide information to policy makers to better allocate health care resources to those with diminished norm-based scores.

**Supplementary Information:**

The online version contains supplementary material available at 10.1186/s12889-024-18391-3.

## Introduction

The EQ-5D is a brief self-completion instrument developed by the EuroQoL group in the 1980s [1]. It has been proven to be a valid and reliable tool for HRQoL measurement in both general population samples and in clinical contexts [2, 3]. As a result, the EQ-5D is commonly used to compute HU scores using country-specific value sets for economic analyses [4, 5]. The first version, EQ-5D-3L, has five questions, one for each of the following dimensions: mobility (MO), self-care (SC), usual activities (UA), pain/discomfort (PD), and anxiety/depression (AD). Each dimension has three response options including no problem, some/moderate problem, and extreme problem [6]. A newer version, EQ-5D-5L, still has five questions by retaining the original five dimensions, but expanding the response options from three to five levels for each dimension as follows: no problem, slight problem, moderate problem, severe problem, and extreme problem/unable to perform. These modifications resulted in the EQ-5D-5L being able to cover a wider range of health states and having enhanced discriminatory power for use in both the general public and in therapeutic settings [2, 3].

EQ-5D-3L population norms have been reported for Japan [7], six European countries (Belgium, France, Germany, Italy, the Netherlands, and Spain) [8], while EQ-5D-5L population norms have been also reported for a number of Asian, European, American, and African countries including South Australia [9], Barbados and Jamaica [10], Belgium [11] Bulgaria [12], Canada [13], China [14], Hong Kong [15], Columbia [16], Denmark [17], England [18], France [19], Germany [20], Indonesia [21], Iran [22], Ireland [23], Japan [24], New Zealand [25], Norway [26], Poland [27], Russia [28], Slovenia [29], Spain [30], Sweden [31], Trinidad and Tobago [32], the United States [33], and Vietnam [34].

In Thailand, the EQ-5D has been widely used to assess and evaluate health interventions, and it has also been strongly recommended for economic analyses by the recent Thai Health Technology Assessment Guidelines [35]. The EQ-5D-5L has been proven to be a practical, reliable, and valid instrument, with better discriminatory power than the EQ-5D-3L in the general population [3] and in therapeutic settings [36, 37]. However, both versions have been used to generate HU scores in patients with chronic diseases, especially for cancer patients [38], because both versions have a Thai-specific value set to compute the HU scores [39, 40]. As a result, there is clear need to establish population norms for the EQ-5D-3L and EQ-5D-5Lto benchmark the HU scores for evaluating population health care, health equity, and health care interventions among the Thai population.

To the best of our knowledge, only one study [41] has been conducted to estimate and report EQ-5D-5L population norms for the general Thai population. However, these population norms were developed based on a sample with a limited age range (20–70 years of age) to be considered representative for a national survey because the samples with some age groups were not included especially for the aged >70 years of age considered a vulnerable group of having diminished HRQoL level for both physical and mental health. Moreover, the previous Thai norms only sampled participants from five provinces of Thailand, which does not adequately represent the whole country, particularly since provinces from metropolitan area were not recruited. Previous evidence revealed that people living in rural and metropolitan areas could have some variations of HRQoL levels due to their different lifestyles, activities, and personal and social characteristics [42]. This is also in contrast to the original valuation study of the EQ-5D-5L in Thailand, where 12 provinces were randomly sampled [39].

Notably, the data for the previous norms [41] were collected during the pre-COVID-19 pandemic. Many studies have investigated the HRQoL measurement during and after the COVID-19 pandemic, and they revealed that people often have experienced diminished physical and mental health after the pandemic particularly due to the prevalence of depressive symptoms post COVID-19 [43–46]. There is a need to update the norms after the COVID-19 pandemic for the Thai population. Therefore, the objectives of this study were to develop and compare the HU norm-based scores obtained from the EQ-5D-3L and EQ-5D-5L with a wider age range (≥ 18 years old) and larger sample size (2,000 respondents) from 12 provinces to improve generalizability of the results and to find the associations between the sociodemographic factors and HU scores. We also aimed to determine the consistency of response redistribution between EQ-5D versions and estimate the disutility (1-HU scores) associated with self-reported main diseases to facilitate QALYs calculation for economic analyses.

## Method

### Study design

A cross-sectional survey study was conducted with participants (*n* = 2,000) from the general Thai population. We specified the region and metropolitan areas to be selected, and 12 provinces were randomly selected specific to each specified regions and area as follows:1) Bangkok Metropolitan (Bangkok, Samut Prakan and Nonthaburi), 2) Central (Chonburi and Nakhon Pathom), 3) North (Chiang Mai and Nakhon Sawan), 4) North-east (Nakhon Ratchasima, Khon Kaen and Buriram), and 5) South (Nakhon Si Thammarat and Phatthalung). A quota sampling method was employed to select the study participants in proportion to age, sex, and area of residence (metropolitan and rural areas) to represent the general Thai population. Face-to-face interviews were conducted with individuals at their home residences from May to June 2023.

### Instruments

#### EQ-5D

The EQ-5D is a questionnaire for measuring current health state to generate HU scores for economic analyses. Each participant is asked to rate their health on the day that the questionnaire is administered. It is comprised of two parts: (1) descriptive system containing five questions for each of the five dimensions: MO, SC, UA, PD, and AD, and (2) EQ-VAS that requires respondents to rate their current health status on a 20-cm vertical line where its endpoints are labeled as worst imaginable health state at 0 and best imaginable health state at 100, resulting in a score range of 0 to 100 [1]. The responses to the descriptive system are used to compute the HU scores using a country-specific value set. At present, both Thai EQ-5D-3L and EQ-5D-5L have their own value set where the maximum score is 1.000 representing perfect health, while the minimum scores are ˗0.4540 and ˗0.4212 for the worst rates health states of the EQ-5D-3L [40] and EQ-5D-5L [39], respectively. Notably, a negative HU score indicates a health state worse than death. The EuroQoL group officially granted to use both versions in this study.

#### Procedure

Individuals who met the pre-defined eligibility criteria were asked to participate in the face-to-face interviews with well-trained interviewers. The eligibility criteria included (1) age ≥ 18 years, (2) understanding of Thai language and data collection process evaluated by the primary researcher or the interviewers. However, we did not recruit participants if they had been diagnosed with an acute or life-threatening illness or had cognitive impairment. The interviewers were instructed to read questions and response options without explaining the meanings to the study participants to ensure that they completed the questionnaires based on their own understanding.

Before the interviews commenced, the participant information sheet was given to each study participant, and written informed consent was obtained to document voluntary participation from each study participant. However, they could withdraw from the study at any time if they felt uncomfortable. Each participant was asked to complete the 23-item questionnaire in the following order: (1) demographic information (12 items), (2) EQ-5D-3L (5 items), (3) EQ-5D-5L (5 items) and (4) EQ-VAS (1 item). The demographic section inquired about the following aspects: sex, age, education level, health insurance scheme, occupation, average monthly household income, health conditions, main disease affecting their health, smoking, alcohol consumption, weight, and height.

### Data analyses

Descriptive statistics (mean, standard deviations [SD], frequencies and percentage) were used to report the sociodemographic characteristics of the study participants where appropriate, and to compare with the sample characteristics from the original valuation EQ-5D-5L study [39]. Frequencies and percentages were used to report the responses for both EQ-5D versions. Furthermore, frequencies and percentages were employed to report the response redistribution determining the response consistency between EQ-5D versions. In order to quantify consistency, the response to EQ-5D-3L was recorded to the EQ-5D-5L response (3L_5L_) as follows: 1 = 1, 2 = 3, and 3 = 5, and inconsistency size was calculated from the responses to both EQ-5D versions as| 3L_5L_– 5L| -1, so zero or less than zero can be determined as consistency for the responses between EQ-5D versions [3, 47]. The mean EQ-VAS scores were also presented for each pair to ensure the validity of response redistribution.

The responses to the EQ-5D-3L and EQ-5D-5Lquestionnaires were converted to HU scores using the Thai value sets for both EQ-5D-3L [40] and EQ-5D-5L [39], while the EQ-VAS scores were reported in the form of 0-100. Due to high ceiling effects of the HU and EQ-VAS scores, non-parametric statistics were employed to test the differences of HU scores derived from those two instruments stratified by demographic factors including Mann-Whitney U test and Kruskal Wallis *H* tests for two and more than two groups categorical variables, respectively. The HU and EQ-VAS scores were also presented and reported by six age bands (< 30, 30–39, 40–49, 50–59, 60–69, and ≥ 70) and stratified by sex. Moreover, the intraclass correlation coefficient (ICC) was used to determine the agreement level of the HU scores from those two instruments. The ICC was computed with the two-way mixed-effects model based on absolute agreement, yielding an ICC ranging from 0.00 to 1.00 [48]. The ICC was classified into four levels of agreements as poor agreement (ICC < 0.50), moderate agreement (0.50 ≤ ICCs < 0.75), good agreement (0.75 ≤ ICCs < 0.90), and excellent agreement (ICCs ≥ 0.90) [48].

Due to highly skewed data for the HU and EQ-VAS scores, the associations between significant factors from the univariate analysis as independent variables and the HU and EQ-VAS scores as dependent variables were investigated using multivariable Tobit regression models [49]. Prior to running the regression analyses, Spearman correlation’s rho was employed to generate a correlation matrix of sociodemographic factors among each other to test the influence of multicollinearity [24]. Any sociodemographic variable with an absolute value of Spearman’s rho > 0.30 was considered as exhibiting collinearity with another variable [24] and were not therefore entered into the Tobit regression model. Furthermore, another Tobit regression analysis was performed to determine the size of the association of disutility value (disutility = 1-HU scores) from two instruments with the self-reported main disease. Moreover, the EQ-VAS scores were converted to HU scores where the EQ-VAS scores were divided by 100 and were used to estimate the size of disutility value similar to other HU scores. All analyses were performed using STATA 17 (StataCorp LLC, College Station, TX, USA), with a *p*-value of < 0.05 being considered statistically significant.

## Results

### Sample characteristics

Table 1 illustrates the sample characteristics of this study. The majority of participants were female (53.0%) with an average age was 46.2 ± 16.9 years, and 67.2% living in a rural area. Compared to the previous EQ-5D-5L valuation study [39], our samples shared the similar proportions of some characteristics in term of sex and some age bands.

**Table 1:** The sample characteristics as compared to those from the original EQ-5D-5L valuation study

Characteristics	Study samples		EQ-5D-5L valuation study [39]
	*n*	%	%
Sex			
Male	940	47.00	48.38
Female	1,060	53.00	51.62
Marital status			
Single	541	27.10	19.14
Married	1,109	55.56	67.61
Widowed	211	10.57	N/A
Divorced/separated	135	6.76	N/A
Mean age (*SD*) in years	46.16 (16.86)		
Age (years)			
< 30	400	20.00	20.80
30–39	371	18.55	21.71
40–49	374	18.70	22.62
50–59	352	17.60	17.23
60–69	304	15.20	N/A
≥ 70	199	9.95	N/A
Health insurance			
Social security	398	19.95	N/A
Universal coverage	1,426	71.48	N/A
Civil servant benefit scheme	156	7.82	N/A
Private health insurance	15	0.75	N/A
Education level			
No or Elementary	627	31.41	44.99
Secondary	807	40.43	44.16
College/University	562	28.16	10.85
Mean monthly household income (SD) in Thai Baht	32,134.23 (23,510.78)		22,602.86 (26,757.98)
Monthly household income (Thai Baht)			
≤ 10,000	148	7.54	N/A
10,001–50,000	1,651	84.11	N/A
50,001-100,000	148	7.54	N/A
> 100,001	16	0.82	N/A
Employment status			
Employed	1,569	78.53	N/A
Unemployed	265	13.26	N/A
Students	132	6.61	N/A
Retired	32	1.60	N/A
Residence of origin			
Municipality	656	32.80	43.33
Rural	1,344	67.20	56.67
Self-reported health condition			
Healthy	1,267	63.35	N/A
Reported health conditions	733	36.65	N/A
Main disease			
Hypertension	465	33.02	N/A
Diabetes	246	33.56	N/A
Dyslipidemia	64	8.73	N/A
Asthma/COPD	18	2.46	N/A
Bone and joint disorders	55	7.50	N/A
Stroke	9	1.23	N/A
Renal failure	19	2.59	N/A
Heart	33	4.50	N/A
Cancer	4	0.55	N/A
Others	43	5.87	N/A
Smoking status			
Smokers	428	21.44	N/A
Non-smokers	1,568	78.56	N/A
Alcohol consumptions			
Drinker	731	36.62	N/A
Non-drinker	1,265	63.38	N/A
Body mass index (mean [SD])	22.82 (3.60)		N/A

N/A denotes that the data were not reported in the original EQ-5D-5L valuation study

### Response distribution and redistribution for both EQ-5D versions

Table 2 shows the response distribution to both EQ-5D versions. The participants rated themselves as no problems with the SC dimension with the highest percentage for both EQ-5D versions (95.50% for the EQ-5D-3L vs. 94.95% for the EQ-5D-5L) followed by MO (84.05% vs. 83.55%), UA (83.40% vs. 81.00%), AD (76.10% vs. 67.55%) and PD (68.95% vs. 61.00%) for both versions. Of note, it also showed that the percentages of reporting “no problems” for all EQ-5D-5L dimensions were lower than those of the EQ-5D-3L dimensions.

**Table 2:** Response distribution to both EQ-5D versions

Instruments	Response options	Frequencies (%)				
		MO	SC	UA	PD	AD
EQ-5D-3L	No problem (Level 1)	1681 (84.05)	1910 (95.50)	1668 (83.40)	1379 (68.95)	1522 (76.10)
	Some/Moderate problem (Level 2)	312 (15.60)	85 (4.25)	322 (16.10)	610 (30.50)	460 (23.00)
	Extreme problem (Level 3)	7 (0.35)	5 (0.25)	10 (0.50)	11 (0.55)	18 (0.90)
EQ-5D-5L	No problem (Level 1)	1671 (83.55)	1899 (94.95)	1620 (81.00)	1220 (61.00)	1351 (67.55)
	Slight problem (Level 2)	233 (11.65)	73 (3.65)	276 (13.80)	531 (26.55)	479 (23.95)
	Moderate problem (Level 3)	85 (4.25)	21 (1.05)	86 (4.30)	227 (11.35)	161 (8.05)
	Severe problem (Level 4)	6 (0.30)	4 (0.20)	13 (0.65)	17 (0.85)	4 (0.20)
	Extreme/Unable to perform (Level 5)	5 (0.25)	3 (0.15)	5 (0.25)	5 (0.25)	5 (0.25)

MO (Mobility), SC (Self-care), UA (Usual Activities), PD (Pain/discomfort), AD (Anxiety/depression)

As shown in Table 3, most of the samples reporting level 1-EQ-5D-3L remained at the level 1-EQ-5D-5L for all EQ-5D dimensions ranging from 86.73% for AD to 98.95% for SC. Similarly, our samples rating level 2- EQ-5D-3L shifted their answers to level 2- EQ-5D-5L from 58.69% for PD to 67.71% for SC, whereas the proportion of samples ranging from 21.18% for SC to 36.72% for PD of the samples upgraded their answers to level 3-EQ-5D-5L. Our samples rated themselves as level 3-EQ-5D-3L redistributed their answers to level 4-EQ-5D-5L ranging from 11.11% for AD to 45.45% for PD, while approximately 27.78% for AD ˗ 71.43% for MO of our samples redistributed their response to level 5-EQ-5D-5L. Of the 10,000 redistribution pairs, some inconsistent pairs were observed in five dimensions: MO (*n* = 16, 0.16%) SC (*n* = 12, 0.12%), UA (*n* = 14, 0.14%), PD (*n* = 19, 0.19%), and AD (*n* = 48, 0.48%).

**Table 3:** Response redistribution from EQ-5D-3L to EQ-5D-5L

EQ-5D-3L	EQ-5D-5L				
Dimensions	Level 1	Level 2	Level 3	Level 4	Level 5
Mobility (MO) n (%^a^) [mean EQ-VAS^b^, size of inconsistency^c^]					
Level 1	1656 (98.51) [82.11, − 1]	25 (1.49) [76.00, 0]	**0 (0.00)** **[0.00, 1]**	**0 (0.00)** **[0.00, 2]**	**0 (0.00)** **[0.00, 3]**
Level 2	**15 (4.81)** **[74.33, 1]**	208 (66.67) [70.07, 0]	84 (26.92) [65.36, − 1]	5 (1.60) [52.00, 0]	**0 (0.00)** **[0.00, 1]**
Level 3	**0 (0.0)** **[0.00, 3]**	**0 (0.0)** **[0.00, 2]**	**1 (14.29)** **[60.00, 1]**	1 (14.29) [30.00, 0]	5 (71.43) [50.00, − 1]
Self-care (SC)					
Level 1	1890 (98.95) [80.68, − 1]	18 (0.94) [69.44, 0]	**2 (0.10)** **[55.00, 1]**	**0 (0.00)** **[0.00, 2]**	**0 (0.00)** **[0.00, 3]**
Level 2	**9 (10.90)** **[65.56, 1]**	55 (67.71) [67.35, 0]	18 (21.18) [59.17, − 1]	3 (3.53) [73.33, 0]	**0 (0.00)** **[0.00, 1]**
Level 3	**0 (0.0)** **[0.00, 3]**	**0 (0.0)** **[0.00, 2]**	**1 (20.00)** **[0.00, 1]**	1 (20.00) [0.00, 0]	3 (60.00) [0.00, − 1]
Usual activities (UA)					
Level 1	1608 (96.40) [82.50, − 1]	59 (3.54) [76.07, 0]	**1 (0.06)** **[100.0, 1]**	**0 (0.00)** **[0.00, 2]**	**0 (0.00)** **[0.00, 3]**
Level 2	**12 (3.73)** **[77.50, 1]**	216 (67.08) [69.11, 0]	85 (26.40) [63.26, − 1]	9 (2.80) [64.44, 0]	**0 (0.00)** **[0.00, 1]**
Level 3	**0 (0.00)** **[0.00, 3]**	**1 (10.00)** **[80.00, 2]**	**0 (0.00)** **[0.00, 1]**	4(40.00) [62.50, 0]	5 (50.00) [53.00, − 1]
Pain/discomfort (PD)					
Level 1	1204 (87.31) [84.79, − 1]	173 (12.55) [78.16, 0]	**2 (0.15)** **[75.00, 1]**	**0 (0.00)** **[0.00, 2]**	**0 (0.00)** **[0.00, 3]**
Level 2	**16 (2.62)** **[72.13, 1]**	358 (58.69) [72.71, 0]	224 (36.72) [68.66, − 1]	12 (1.97) [68.33, 0]	**0 (0.0)** **[0.00, 1]**
Level 3	**0 (0.00)** **[0.00, 3]**	**0 (0.00)** **[0.00, 2]**	**1 (9.09)** **[30.00, 1]**	5 (45.45) [52.00, 0]	5 (45.45) [45.00, − 1]
Anxiety/depression (AD)					
Level 1	1320 (86.73) [82.95, − 1]	192 (12.61) [78.56, 0]	**10 (0.66)** **[86.50, 1]**	**0 (0.00)** **[0.00, 2]**	**0 (0.00)** **[0.00, 3]**
Level 2	**27 (5.87)** **[81.59, 1]**	284 (61.74) [72.19, 0]	147 (31.96) [68.27, − 1]	2 (0.43) [85.00, 0]	**0 (0.0)** **[0.00, 1]**
Level 3	**4 (22.22)** **[82.50, 3]**	**3 (16.67)** **[78.33, 2]**	**4 (22.22)** **[92.50, 1]**	2 (11.11) [55.00, 0]	5 (27.78) [53.00, − 1]

Inconsistencies are presented in bold

^a^ Percentage in each level of the EQ-5D-3L

^b^ Mean of EQ-VAS from the EQ-5D-5L

^c^ Size of inconsistent response for each pair

### Population norms of the EQ-5D-3L, EQ-5D-5L, and EQ-VAS

Table 4 shows the Thai population norms of the HU scores from two instruments classified by sex and age bands. As expected, the HU scores and the percentage of respondents with full health were generally higher for the younger age band (< 30 years) than other age bands. Moreover, male participants had higher EQ-5D-3L, EQ-5D-5L, and EQ-VAS scores than females for most age groups except for the participants aged < 30 years. For example, for participants aged < 30 years, the mean HU score for the EQ-5D-3L was 0.949 for males and 0.953 for females, for the EQ-5D-5L 0.972 (male) and 0.976 (female). For respondents aged ≥ 70 years, in contrast, those scores were 0.589 (male) and 0.560 (female) for the EQ-5D-3L, 0.774 (male) and 0.764 (female) for the EQ-5D-5L.

**Table 4:** Population norms of the EQ-5D-3L, EQ-5D-5L and EQ-VAS

Instruments	Age	Sex	*n*	Mean	*SD*	Median	Interquartile range	% full health
EQ-5D-3L	< 30	Male	201	0.949	0.111	1.000	(1.000,1.000)	81.59
		Female	199	0.953	0.102	1.000	(1.000,1.000)	81.91
	30–39	Male	189	0.940	0.119	1.000	(1.000,1.000)	78.84
		Female	182	0.915	0.132	1.000	(0.766,1.000)	69.78
	40–49	Male	176	0.908	0.152	1.000	(0.766,1.000)	70.45
		Female	198	0.896	0.154	1.000	(0.766,1.000)	66.16
	50–59	Male	164	0.838	0.179	1.000	(0.677,1.000)	52.44
		Female	188	0.804	0.183	0.726	(0.640,1.000)	43.62
	60–69	Male	132	0.779	0.202	0.726	(0.635,1.000)	40.91
		Female	172	0.714	0.222	0.677	(0.573,1.000)	30.23
	≥ 70	Male	78	0.589	0.228	0.573	(0.425,0.677)	14.10
		Female	121	0.560	0.262	0.573	(0.425,0.677)	10.74
	Total	-	2000	0.845	0.204	1.000	(0.694,1.000)	57.80
EQ-5D-5L	< 30	Male	201	0.972	0.050	1.000	(0.944,1.000)	70.15
		Female	199	0.976	0.044	1.000	(0.942,1.000)	73.87
	30–39	Male	189	0.968	0.052	1.000	(0.942,1.000)	66.14
		Female	182	0.964	0.054	1.000	(0.942,1.000)	61.54
	40–49	Male	176	0.953	0.077	1.000	(0.942,1.000)	60.80
		Female	198	0.954	0.071	1.000	(0.934,1.000)	58.59
	50–59	Male	164	0.926	0.088	0.944	(0.878,1.000)	44.51
		Female	188	0.903	0.097	0.942	(0.827,1.000)	32.45
	60–69	Male	132	0.893	0.109	0.934	(0.827,1.000)	32.58
		Female	172	0.865	0.127	0.885	(0.816,1.000)	23.84
	≥ 70	Male	78	0.774	0.173	0.814	(0.703,0.878)	6.41
		Female	121	0.764	0.192	0.796	(0.703,0.878)	8.26
	Total	-	2000	0.923	0.113	0.943	(0.885,1.000)	49.05
EQ-VAS	< 30	Male	201	85.37	9.66	90.00	(80.00,90.00)	7.96
		Female	199	86.94	9.25	90.00	(80.00,95.00)	5.03
	30–39	Male	189	84.26	8.92	85.00	(80.00,90.00)	3.17
		Female	182	82.12	10.17	80.00	(80.00,90.00)	3.30
	40–49	Male	176	81.77	9.24	80.00	(77.50,90.00)	2.27
		Female	198	82.47	10.12	80.00	(80.00,90.00)	3.54
	50–59	Male	164	78.46	10.50	80.00	(70.00,85.00)	2.44
		Female	188	76.72	11.05	77.50	(70.00,85.00)	2.13
	60–69	Male	132	77.25	10.91	80.00	(70.00,85.00)	2.27
		Female	172	73.73	11.67	70.00	(70.00,80.00)	1.16
	≥ 70	Male	78	67.97	12.48	70.00	(60.00,80.00)	1.28
		Female	121	67.25	12.11	68.00	(60.00,75.00)	0.83
	Total	-	2000	79.83	11.75	80.00	(70.00,90.00)	3.20

Regarding the HU scores from the two instruments, we found that the EQ-5D-5L yielded the highest HU scores for all sexes and age bands. Nevertheless, the highest percentage of participants with perfect health was observed for the EQ-5D-3L followed by the EQ-5D-5L. The ICC between the HU scores was 0.789 (95% CI: 0.558–0.878). for EQ-5D-3L/EQ-5D-5L pair.

**Fig. 1:**
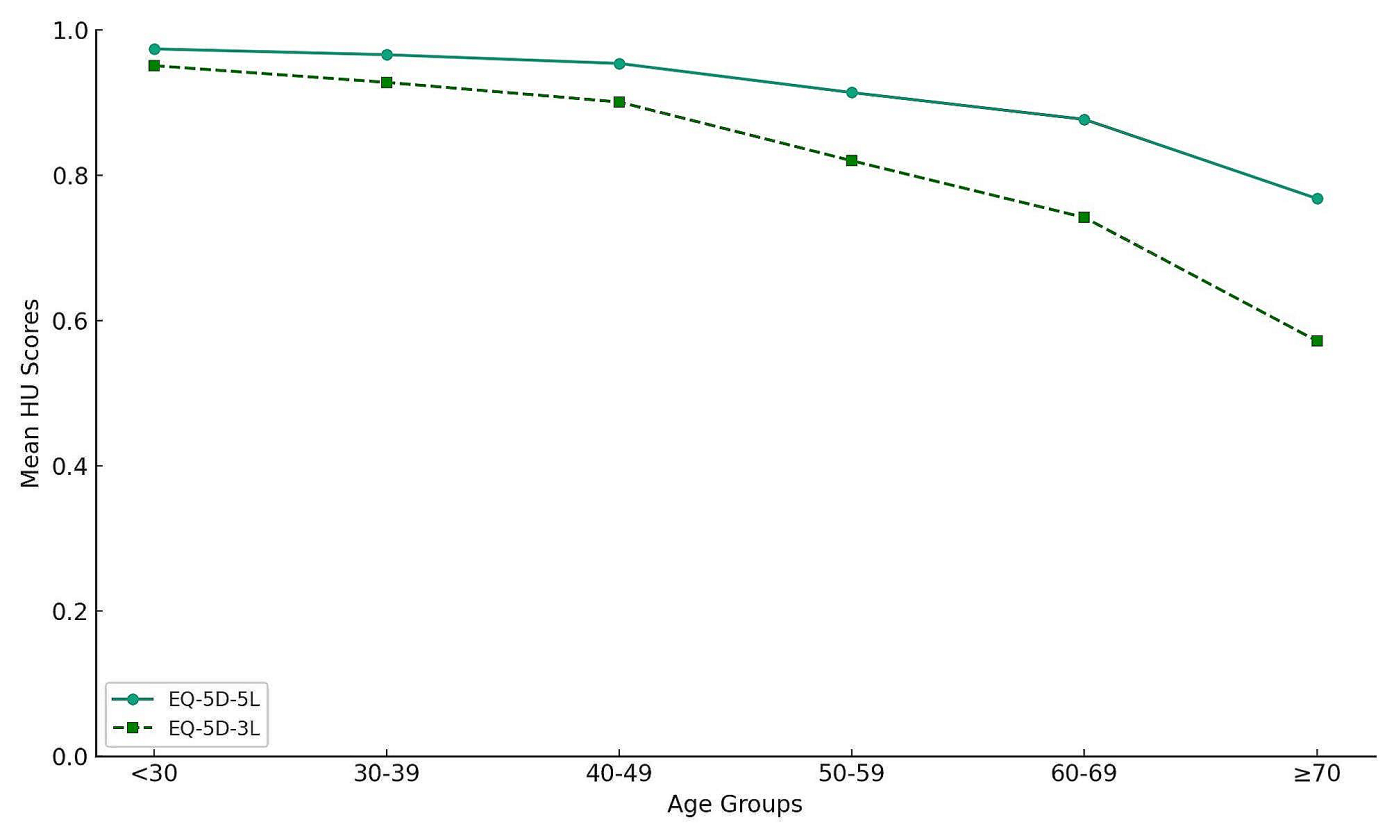
Comparison of population norms between EQ-5D-3L and EQ-5D-5L

As shown in Fig. 1, the mean EQ-5D-5L scores were higher than those of the EQ-5D-3L scores for all sexes and age bands (*p* < 0.05). The responses to those two instruments are presented in the Supplementary materials.

### Relationships between norms-based scores and sociodemographic information

Table 5 conveys the results of univariate analyses between norms-based scores and sociodemographic factors. The mean EQ-5D-3L and EQ-5D-5L were 0.845 (*SD* = 0.204) and 0.923 (*SD* = 0.113). The univariate analysis showed that female and older respondents had significantly (*p* < 0.05) lower HU scores for those two instruments compared to males and younger participants. Moreover, the HU scores from the two instruments were affected by other sociodemographic factors including marital status, education level, health insurance scheme, monthly household income, employment status, self-reported health conditions, smoking, alcohol consumptions, and BMI (all *p* < 0.05). The mean EQ-VAS score was 79.83 (*SD* = 11.75) as also shown in Table 5. Similar to the HU scores, it was generally reduced for female and older participants, and it was affected by other sociodemographic factors, as well (all *p* < 0.05).

**Table 5:** EQ-5D-3L, EQ-5D-5L and EQ-VAS population norms by sample characteristics

Characteristics	*n*	EQ-5D-3L			EQ-5D-5L			EQ-VAS		
		Mean	Standard deviation	*p*-value	Mean	Standard deviation	*p*-value	Mean	Standard deviation	*p*-value
**Overall**	2000	0.845	0.204	N/A	0.923	0.113	N/A	79.83	11.75	N/A
**Sex**										
Male	940	0.866	0.189	< 0.001	0.932	0.103	< 0.001	80.68	11.12	0.007
Female	1060	0.826	0.216		0.915	0.121		79.07	12.24	
**Marital status**										
Single	541	0.940	0.119	< 0.001	0.972	0.049	< 0.001	85.06	10.12	< 0.001
Married	1109	0.840	0.191		0.922	0.100		79.17	10.94	
Widow	211	0.632	0.266		0.804	0.188		70.47	13.12	
Divorced/Seperated	135	0.834	0.213		0.917	0.106		78.81	11.33	
**Age**										
<30	400	0.951	0.106	< 0.001	0.974	0.047	< 0.001	86.15	9.48	< 0.001
30–39	371	0.928	0.126		0.966	0.053		83.21	9.60	
40–49	374	0.901	0.153		0.954	0.074		82.14	9.71	
50–59	352	0.820	0.182		0.914	0.094		77.53	10.81	
60–69	304	0.742	0.215		0.877	0.120		75.26	11.46	
≥70	199	0.572	0.249		0.768	0.184		67.53	12.23	
**Health insurance**										
Social security	398	0.914	0.144	< 0.001	0.958	0.075	< 0.001	83.69	10.29	< 0.001
Universal coverage	1426	0.827	0.214		0.913	0.119		78.44	11.84	
Civil servant benefit schemes	156	0.830	0.212		0.922	0.124		82.85	11.76	
Private health insurance	15	0.932	0.119		0.960	0.047		84.27	8.12	
**Education levels**										
No or Elementary	627	0.719	0.237	< 0.001	0.858	0.148	< 0.001	73.16	12.11	< 0.001
Secondary	807	0.893	0.165		0.945	0.085		81.34	10.37	
College/University	562	0.916	0.144		0.963	0.062		84.99	9.61	
**Monthly household income**										
≤ 10,000	148	0.775	0.247	< 0.001	0.887	0.162	< 0.001	73.76	12.77	< 0.001
10,001–50,000	1651	0.846	0.203		0.923	0.111		80.01	11.56	
50,001-100,000	148	0.903	0.157		0.956	0.070		83.80	10.77	
>100,001	16	0.756	0.155		0.903	0.081		75.31	11.32	
**Employment status**										
Employed	1569	0.874	0.170	< 0.001	0.941	0.083	< 0.001	80.74	10.69	< 0.001
Unemployed	265	0.636	0.267		0.802	0.179		70.65	13.61	
Students	132	0.960	0.097		0.977	0.042		88.27	8.97	
Retired	32	0.666	0.666		0.816	0.184		76.25	11.43	
**Residence of origin**										
Municipality	656	0.851	0.205	0.299	0.930	0.103	0.007	80.81	11.35	0.015
Rural	1344	0.842	0.204		0.919	0.118		79.35	11.92	
**Self-reported health conditions**										
Healthy	1267	0.929	0.129	< 0.001	0.967	0.056	< 0.001	83.89	9.54	< 0.001
Reported health conditions	733	0.699	0.227		0.847	0.143		72.82	11.93	
**Smoking status**										
Smokers	428	0.896	0.159	< 0.001	0.951	0.076	< 0.001	82.27	10.01	< 0.001
Non-smokers	1568	0.831	0.213		0.915	0.120		79.18	12.11	
**Alcohol consumptions**										
Drinkers	731	0.908	0.147	< 0.001	0.959	0.070	< 0.001	83.02	9.99	< 0.001
Non-drinkers	1265	0.808	0.223		0.902	0.128		78.01	12.30	
**Body mass index**										
< 18.5	192	0.891	0.168	< 0.001	0.940	0.092	< 0.001	82.90	11.52	< 0.001
18.5–22.9	944	0.853	0.202		0.927	0.115		80.16	11.54	
23.0-24.9	400	0.841	0.207		0.924	0.104		79.89	11.04	
25.0-29.9	375	0.834	0.198		0.923	0.101		79.09	11.68	
≥ 30	77	0.714	0.269		0.834	0.182		72.35	15.06	

Table 6 depicts the relationships between the HU scores and sociodemographic factors using Tobit regression models. Analysis 1 included sex, age bands, monthly household income, and smoking as predictor variables which had significant association with the HU and EQ-VAS scores in the previous Thai population norms study [41]. HU scores for all two instruments were associated with age band (all *p* < 0.05). However, for sex, only EQ-5D-3L and EQ-5D-5L scores were significant predictors (both *p* < 0.05). Analysis 2 included all significant demographic factors identified from the univariate analysis. Seven sociodemographic factors, including sex, age band, average monthly household income, occupations, residence of origin, health insurance, and BMI, were employed to investigate the associations with HU scores because their absolute values of Spearman correlation were less than 0.3. Similar to the analysis 1, only age band was associated with HU and EQ-VAS scores. Compared with the samples aged < 30 years, older samples had lower mean HU and EQ-VAS scores where the greatest difference was found among the oldest age group (≥ 70 years). Unlike the analysis 1, female samples reported lower mean HU and EQ-VAS scores than male samples; however, significant difference was only found with EQ-5D-3L scores (*β* = -0.045, *p* = 0.010). Regression also showed that unemployed and obese (BMI ≥ 30) participants reported lower HU and EQ-VAS scores than their counterparts (all *p* < 0.05).

**Table 6:** Relationship between norm-based scores from EQ-5D-3L, EQ-5D-5L, EQ-VAS and sample characteristics

Variables	Analysis 1				Analysis 2			
	**EQ-5D-3L**		**EQ-5D-5L**		EQ-5D-3L		EQ-5D-5L	
	Coefficient	*p*-value	Coefficient	*p*-value	Coefficient	*p*-value	Coefficient	*p*-value
**Sex (Ref: Male)**								
Female	-0.057	0.001	-0.017	0.030	-0.045	0.010	-0.009	0.227
**Age group (Ref: <30)**								
30–39	-0.093	0.004	-0.032	0.019	-0.074	0.037	-0.030	0.048
40–49	-0.158	< 0.001	-0.056	< 0.001	-0.146	< 0.001	-0.058	< 0.001
50–59	-0.327	< 0.001	-0.135	< 0.001	-0.298	< 0.001	-0.126	< 0.001
60–69	-0.448	< 0.001	-0.187	< 0.001	-0.407	< 0.001	-0.167	< 0.001
≥70	-0.670	< 0.001	-0.316	< 0.001	-0.572	< 0.001	-0.261	< 0.001
**Health insurance (Ref: Social security)**								
Universal coverage					0.001	0.969	0.001	0.949
Civil servant benefit scheme					-0.020	0.612	0.004	0.802
Private health insurance					0.104	0.370	0.003	0.951
**Monthly household income in Thai Baht (Ref: ≤ 10,000)**								
10,001–50,000	0.001	0.989	< 0.001	0.999	-0.015	0.643	-0.012	0.388
50,001-100,000	0.068	0.139	0.037	0.073	0.053	0.267	0.018	0.393
>100,001	-0.191	0.035	-0.048	0.263	-0.207	0.022	-0.064	0.125
**Employment status (Ref: Employed)**								
Unemployed					-0.128	< 0.001	-0.082	< 0.001
Students					0.055	0.285	0.006	0.762
Retired					-0.050	0.440	-0.053	0.075
**Residence of origin (Ref: Municipality)**								
Rural					-0.017	0.360	-0.025	0.003
**Smoking (Ref: Non-smokers)**								
Smokers								
**Body mass index (Ref: < 18.5)**								
18.5–22.9					-0.019	0.566	0.005	0.747
23.0-24.9					0.013	0.727	0.014	0.393
25.0-29.9					0.014	0.716	0.017	0.315
≥30.0					-0.166	0.001	-0.100	< 0.001
	**EQ-VAS**				**EQ-VAS**			
	**Coefficient**		***p*** **-value**		**Coefficient**		***p*** **-value**	
**Sex (Ref: Male)**								
Female	-0.704		0.194		-0.803		0.096	
**Age group (Ref: <30)**								
30–39	-2.952		< 0.001		-1.718		0.045	
40–49	-3.986		< 0.001		-2.894		0.001	
50–59	-8.473		< 0.001		-6.667		< 0.001	
60–69	-10.545		< 0.001		**-**8.572		< 0.001	
≥70	-18.308		< 0.001		-15.218		< 0.001	
**Health insurance (Ref: Social security)**								
Universal coverage					-1.768		0.007	
Civil servant benefit scheme					1.856		0.079	
Private health insurance					0.776		0.780	
**Monthly household income in Thai Baht (Ref: ≤ 10,000)**								
10,001–50,000	2.952		0.001		1.889		0.040	
50,001-100,000	5.665		< 0.001		3.202		0.014	
>100,001	-0.788		0.777		-3.435		0.213	
**Employment status (Ref: Employed)**								
Unemployed					-2.259		0.006	
Students					4.904		< 0.001	
Retired					-0.028		0.988	
**Residence of origin (Ref: Municipality)**								
Rural					-1.052		0.039	
**Smoking (Ref: Non-smokers)**								
Smokers	0.957		0.151					
**Body mass index (Ref: < 18.5)**								
18.5–22.9					-5.780		0.510	
23.0-24.9					0.742		0.450	
25.0-29.9					0.630		0.529	
≥30.0					-6.050		< 0.001	

### Disutility values associated with self-reported main diseases

Table 7 presents disutility values obtained from two instruments associated with self-reported main diseases. Results showed that the pattern of disutility values obtained from two instruments was similar across diseases where bone/joint, stroke, renal, asthma/COPD, produced higher disutility than other diseases. However, differences in disutility values from the two instruments were observed. The disutility values from the EQ-VAS and those two instruments also shared similar patterns across diseases; however, participants with allergic rhinitis did not report significant differences in disutility value as compared to the healthy state.

**Table 7:** Self-reported main disease associated with disutility derived from EQ-5D-3L, EQ-5D-5L and EQ-VAS

Variables	*n*	EQ-5D-3L		EQ-5D-5L		EQ-VAS	
		Coefficient	*p*-value	Coefficient	*p*-value	Coefficient	*p*-value
**Intercept**		0.032	< 0.001	0.019	< 0.001	0.132	< 0.001
**Sex (Ref: Male)**							
Female	1060	0.021	0.003	0.007	0.064	0.006	0.148
**Age group (Ref: <30)**							
30–39	371	0.014	0.226	0.003	0.646	0.025	< 0.001
40–49	374	0.025	0.028	0.007	0.257	0.029	< 0.001
50–59	352	0.062	< 0.001	0.023	0.001	0.055	< 0.001
60–69	304	0.116	< 0.001	0.047	< 0.001	0.066	< 0.001
≥70	199	0.250	< 0.001	0.136	< 0.001	0.125	< 0.001
**Self-reported main disease (Ref: Healthy)**							
Hypertension	242	0.076	< 0.001	0.048	< 0.001	0.042	< 0.001
Diabetes	246	0.145	< 0.001	0.079	< 0.001	0.064	< 0.001
Dyslipidemia	64	0.109	< 0.001	0.042	< 0.001	0.028	0.031
Asthma/COPD	18	0.210	< 0.001	0.102	< 0.001	0.129	< 0.001
Bone/Joint disorders	55	0.292	< 0.001	0.167	< 0.001	0.125	< 0.001
Stroke	9	0.287	< 0.001	0.171	< 0.001	0.091	0.007
Renal disease	19	0.181	< 0.001	0.085	< 0.001	0.142	< 0.001
Heart disease	33	0.153	< 0.001	0.059	< 0.001	0.088	< 0.001
Allergic rhinitis	10	0.146	0.003	0.066	0.020	0.027	0.381
Migraine	7	0.172	0.004	0.088	0.009	0.140	< 0.001
Other diseases	30	0.264	< 0.001	0.134	< 0.001	0.137	< 0.001

## Discussion

The present study generated updated post-COVID Thai norms-based HU scores for the EQ-5D-3L and the EQ-5D-5Lwith a larger general Thai population to improve generalizability of the norm-based scores. We also established the data set of disutility value specific to main disease obtained from those two instruments and EQ-VAS to facilitate HU score calculations for participants with particular diseases in Thailand. However, this study did not report the disutility value for co-morbidities. Therefore, for participants reporting several chronic diseases such as those with hypertension, diabetes, and dyslipidemia, only the main reported disease was analyzed for its effect on the participants’ overall health. Moreover, the disutility value in this study is based on participants residing at their own residence, so generalizability to participants with other conditions is limited such as hospital-based populations.

The percentage of participants reporting “no problem” in each EQ-5D dimension for both EQ-5D-3L and EQ-5D-5L can be ranked as SC (95.5% vs. 95.0%) having the highest percentage, followed by MO (84.1% vs. 83.6%), UA (83.4% vs. 81.0%), AD (76.1% vs. 67.6%), and PD (69.0% vs. 61.0%). This finding implies that the participants were more likely to report problems in mental health for those two instruments (AD for both EQ-5D versions) than physical health (MO and UA for both EQ-5D versions). Similar to earlier work in Mainland China [50] and Japan [7], results of the present study indicated that participants had more impaired mental health than physical health according to both EQ-5D versions. Of note, we found that younger participants (aged < 30 years) reported themselves as having more problems in mental health than physical health from the two instruments as shown in the Supplementary Material. This result is also consistent with those from the previous Thai norms-based study where samples aged < 25 years reported more health problems in mental health (AD) than physical health (MO) of the EQ-5D-5L questionnaire [41]. We reasoned that younger individuals might experience stress due to their work life and social environment [51]. Additionally, if they are experiencing fewer physical health issues due to their younger age, then mental health issues are a more serious consideration in relative terms. Similar to the previous Thai study [3], most of the samples rating themselves at level 1-EQ-5D-3L retained their answers at level 1- EQ-5D-5L for all five dimensions, yielding the presence of high ceiling effects for both EQ-5D versions. These findings indicate that they were relatively healthy which might be due to the exclusion of the samples with acute or life-threatening disease from our study. Nevertheless, some inconsistent responses for both EQ-5D versions were observed with an average proportion at 1.09%, highest and lowest proportions in AD (0.48%) and SC (0.12%), respectively. Therefore, our samples responded to both EQ-5D versions consistently congruent with the reports from previous studies [3, 52].

Regarding the norm-based scores, our results showed that the EQ-5D-5L yielded the mean HU scores of 0.923, while the EQ-5D-3L produced the lower mean HU scores of 0.845 (mean difference = 0.078). Similar to earlier work on head to head comparison for both EQ-5D versions among general Thai population [3, 41] and patients with locally advanced cervical cancer [53], it reported mean difference of 0.08 (0.93 for the EQ-5D-5L and 0.85 for the EQ-5D-3L) and 0.12 (0.755 for the EQ-5D-3L and 0.875 for the EQ-5D-5L) which were close values to our study. We reasoned that the mean differences might be due to different techniques employed to generate the algorithm to calculate the HU scores in that the EQ-5D-3L employed time-trade off [40], while the EQ-5D-5L used both time-trade off and discrete choice experiment techniques (Hybrid method) [39].

As expected, the HU scores from EQ-5D-3L and EQ-5D-5L had a good agreement with the highest ICC of 0.789. Our study showed that the highest ceiling effect was for the EQ-5D-3L scores (57.80%) followed by the EQ-5D-5L (49.05%). These ceiling effects for both EQ-5D versions were similar to those of Asian countries including Japan (68% for the EQ-5D-3L and 55% for the EQ-5D-5L) [7] and Mainland China (urban population 54% for the EQ-5D-5L) [14]; however, those values were higher than those of the western countries such as Switzerland (French speaking population 41.80%) [54] for the EQ-5D-3L and Poland (38.5%) [27], Norway (32.2%) [26], the USA (31.2%) [33] and Germany (30.6%) for the EQ-5D-5L [20]. We reasoned that variations between Asian and Western ethnicities towards EQ-5D items might be accounted for this discrepancy. Nevertheless, this present study and previous studies can confirm that the ceiling effect of the EQ-5D-3L could be reduced when adding two more levels of the response options to the EQ-5D questionnaire. Of note, our ceiling effects for both EQ-5D versions were also close to those of previous Thai study [3] (57.17% for the EQ-5D-3L and 49.08% for the EQ-5D-5L). No floor effects were observed for the EQ-5D-3L and EQ-5D-5L.

Although the HU scores were different across those two instruments, the regression results showed that they consistently declined when increasing age in both analysis 1 and 2. This pattern is similar to the previous norm-based scores from Thailand [41], Iran [22], Vietnam [34] for the EQ-5D-5L and Japan for those two instruments [7] although the age bands were classified differently across studies. Moreover, the present study revealed that unemployed participants and those with BMI ≥ 30 reported lower HU scores from those two instruments than their counterparts. In line with the previous Japanese norms-based study [7] and Hong Kong [15], both analysis 1 and 2 showed that females in the present study reported lower HU scores from those two instruments than male participants although there was no statistical significance for the HU scores from the EQ-5D-5L in analysis 2. Our regression results showed that the HU score difference between both sexes for the EQ-5D-5L (*β* = 0.009) was also close to the those of previous work for the EQ-5D-5L in Japan (*β* = 0.015) [7] and Hong Kong (*β* = 0.005) [15]. Therefore, it could imply that females generally report lower HU scores than males from both EQ-5D versions across populations from different countries.

In relation to the EQ-VAS, our findings indicate that it exhibited a minimal ceiling effect at 3.20%. This figure is notably less than that reported in prior Thai studies, where it was 12.6% [41]. One plausible explanation for this reduced percentage could be the impact of the post-COVID-19 pandemic, which has the potential to deteriorate levels of HRQoL [45]. Similar to other HU scores, the EQ-VAS scores declined with the participants with advancing age. However, some variations for other sociodemographic factors affecting both EQ-VAS and other HU scores were observed. Similar to the previous studies in Thailand [41] and Vietnam [34], the mean EQ-VAS score was 86.06 for the participants achieving the maximum scores of EQ-5D-5L scores (1.000). These findings imply that the participants may consider other health aspects beyond the EQ-5D dimensions when rating their current health with the EQ-VAS, which deserves further investigation in future studies.

Regarding the EQ-VAS scores, we found that the overall difference for mean EQ-VAS scores between this current study and the original valuation EQ-5D-5L valuation study was 3.25 (79.83 vs. 83.08) [39]. Similar to the previous study [55], it reported that the EQ-VAS scores in the post-COVID-19 pandemic were diminished by 8.4% from the pre-pandemic. Furthermore, the response distribution patterns in the EQ-5D dimensions showed changes between the current and original valuation EQ-5D-5L. In the current study, there was a higher percentage of respondents reporting moderate or greater problems than participants in the original valuation EQ-5D-5L study for most of the EQ-5D dimensions. These findings of altered HRQoL levels confirm the rationale of the present study to establish updated population norms for both EQ-5D versions after the COVID-19 pandemic. Nevertheless, the different response patterns to each EQ-5D dimension before and after the COVID pandemic should be further investigated using Rasch analysis based on the item response theory (IRT) in future studies. This could explore the presence of different interpretation of items by different groups and also to what extent items may have shifted in level of difficulty such that they may have become easier or more difficult to endorse.

Similar to previous studies of Japanese population norms for the EQ-5D-3L and EQ-5D-5L [7, 24], bone/joint disorder and stroke yielded the large disutility value (> 0.25) from the two instruments because both diseases could affect the functional capacity and limitation which could contribute to impaired HRQoL [56, 57].

There are some limitations that should be noted. First, the response rate for the interviews was not counted; however, we kept it as maximum as possible due to performing the data collection at the participant’s residence. Second, since this population norm was conducted at the participant’s residence, the results might not be generalizable to other populations such as hospital-based populations. Third, the majority of participants were healthy (63.35%) because we excluded those with acute or life-threatening diseases from this study. Fourth, the disutility value was obtained from those who reported having one main disease, which means that the values of individuals with multiple health conditions were not estimated. Fifth, data validity could be affected by the outline of the survey where the EQ-5D-3L was presented before the EQ-5D-5L. The latter questionnaire inquired about the same health dimensions with different levels of health impairments. Therefore, participants may have been more likely to rate the EQ-5D-5L with the same response of what they had rated themselves on the EQ-5D-3L. Future research needs to explore the possibility of such context effects. Sixth, this study did not report on data for other instruments such as SF-6D because the SF-6D does not have a Thai-specific value set. Future studies could estimate population norms for the SF-6D when the Thai specific-value set is available.

## Conclusion

The present study established updated, post-COVID Thai population norm-based scores from EQ-5D-3L, EQ-5D-5L, and EQ-VAS using a population survey that was larger than previous work for these instruments in Thailand and that also used a larger age range. This population norms are presented the HU scores across sexes and age bands. Results showed that the mean EQ-5D-3L, EQ-5D-5L and EQ-VAS scores were 0.845, 0.923 and 79.83, respectively. Although there were differences in HU scores derived from those instruments, all HU scores have similar trend in terms of decreasing with advancing age and being lower among female, unemployed, and obese (BMI ≥ 30) participants. Regression analyses also showed that bone/joint disorder and stroke contributed to the largest disutility value (> 0.25). In addition, these population norms imply that the mental health among younger adults in Thailand (age < 30 years) deteriorated after the COVID-19 pandemic. The reason for this shift deserves further investigation, such as whether it may be due to the disease and disease control measures during the pandemic. Therefore, it is important to consider the long-term health policy implemented to enhance mental health especially for the younger adults in Thailand after the COVID-19 pandemic.

### Electronic supplementary material

Below is the link to the electronic supplementary material.

Supplementary Material 1

## Data Availability

The datasets used and/or analysed during the current study are available from the corresponding author upon reasonable request.

## Abbreviations

AD: Anxiety or depression
EQ-5D: EuroQoL-5 dimensions
EQ-VAS: EuroQol visual analog scale
HRQoL: Health-related quality of life
HU: Health utility
IQR: Interquartile range
MO: Mobility
PD: Pain or discomfort
SC: Self-care
UA: Usual activities
