# Supplementary material for: EQ-5D-3L and EQ-5D-5L population norms for Thailand
Source: BMC Public Health. 2024 Apr 22;24:1108. doi: 10.1186/s12889-024-18391-3 (PMC11036570; doi:10.1186/s12889-024-18391-3)
Supplement: Supplementary file 1 — Supplementary Material 1 [file 12889_2024_18391_MOESM1_ESM.docx]

**Supplement 1**: Responses of EQ-5D-3L

|  |  |  | Level; n (%) | | | | |
| --- | --- | --- | --- | --- | --- | --- | --- |
| Age bands |  | Dimensions | 1 | 2 | 3 | 4 | 5 |
| <30 |  | Mobility | 394 (98.50) | 6  (1.50) |  |  |  |
|  |  | Self-care | 400  (100) |  |  |  |  |
|  |  | Usual activities | 396  (99.00) | 4  (1.00) |  |  |  |
|  |  | Pain/discomfort | 370  (92.50) | 30  (7.50) |  |  |  |
|  |  | Anxiety/depression | 345  (86.25) | 55  (13.75) |  |  |  |
| 30-39 |  | Mobility | 359  (96.77) | 12  (3.23) |  |  |  |
|  |  | Self-care | 371  (100) |  |  |  |  |
|  |  | Usual activities | 367  (98.92) | 4  (1.08) |  |  |  |
|  |  | Pain/discomfort | 320  (86.25) | 51  (13.75) |  |  |  |
|  |  | Anxiety/depression | 306  (82.48) | 64  (17.25) | 1  (0.27) |  |  |
| 40-49 |  | Mobility | 358  (95.72) | 16  (4.28) |  |  |  |
|  |  | Self-care | 372  (99.47) | 2  (0.53) |  |  |  |
|  |  | Usual activities | 354  (94.65) | 20  (5.35) |  |  |  |
|  |  | Pain/discomfort | 304  (81.28) | 69  (18.45) |  |  |  |
|  |  | Anxiety/depression | 296  (79.14) | 70  (18.72) | 8  (2.14) |  |  |
| 50-59 |  | Mobility | 298  (84.66) | 54  (15.34) |  |  |  |
|  |  | Self-care | 347  (98.58) | 5  (1.42) |  |  |  |
|  |  | Usual activities | 274  (77.84) | 77  (21.88) | 1  (0.28) |  |  |
|  |  | Pain/discomfort | 196  (55.68) | 156  (44.32) |  |  |  |
|  |  | Anxiety/depression | 266  (75.57) | 85  (24.15) | 1  (0.28) |  |  |
| 60-69 |  | Mobility | 209  (68.75) | 95  (31.25) |  |  |  |
|  |  | Self-care | 276  (90.79) | 28  (9.21) |  |  |  |
|  |  | Usual activities | 195  (64.14) | 108  (35.53) | 1  (0.33) |  |  |
|  |  | Pain/discomfort | 140  (46.05) | 161  (52.96) | 3  (0.99) |  |  |
|  |  | Anxiety/depression | 198  (65.13) | 101  (33.22) | 5  (1.64) |  |  |
| ≥70 |  | Mobility | 63  (31.66) | 129  (64.82) | 7  (3.52) |  |  |
|  |  | Self-care | 144  (72.36) | 50  (25.13) | 5  (2.51) |  |  |
|  |  | Usual activities | 82  (41.21) | 109  (54.77) | 8  (4.02) |  |  |
|  |  | Pain/discomfort | 49  (24.62) | 143  (71.86) | 7  (3.52) |  |  |
|  |  | Anxiety/depression | 111  (55.78) | 85  (42.71) | 3  (1.51) |  |  |

**Supplement 2**: Responses of EQ-5D-5L

|  |  |  | Level; n (%) | | | | |
| --- | --- | --- | --- | --- | --- | --- | --- |
| Age bands |  | Dimensions | 1 | 2 | 3 | 4 | 5 |
| <30 |  | Mobility | 395  (98.75) | 5  (1.25) |  |  |  |
|  |  | Self-care | 400  (100) |  |  |  |  |
|  |  | Usual activities | 391  (97.75) | 9  (2.25) |  |  |  |
|  |  | Pain/discomfort | 341  (82.25) | 47  (11.75) | 12  (3.00) |  |  |
|  |  | Anxiety/depression | 312  (78.00) | 64  (16.00) | 24  (6.00) |  |  |
| 30-39 |  | Mobility | 358  (96.50) | 13  (3.50) |  |  |  |
|  |  | Self-care | 370  (99.73) | 1  (0.27) |  |  |  |
|  |  | Usual activities | 361  (97.30) | 10  (2.70) |  |  |  |
|  |  | Pain/discomfort | 289  (77.90) | 62  (16.71) | 20  (5.39) |  |  |
|  |  | Anxiety/depression | 279  (75.20) | 68  (18.33) | 24  (6.47) |  |  |
| 40-49 |  | Mobility | 357  (95.45) | 14  (3.74) | 3  (0.80) |  |  |
|  |  | Self-care | 372  (99.47) | 2  (0.53) |  |  |  |
|  |  | Usual activities | 350  (93.58) | 16  (4.28) | 7  (1.87) | 1  (0.27) |  |
|  |  | Pain/discomfort | 280  (74.81) | 63  (16.84) | 27  (7.22) | 3  (0.80) | 1  (0.27) |
|  |  | Anxiety/depression | 266  (71.12) | 71  (18.98) | 34  (9.09) | 1  (0.27) | 2  (0.53) |
| 50-59 |  | Mobility | 297  (84.38) | 38  (10.80) | 16  (4.55) | 1  (0.28) |  |
|  |  | Self-care | 342  (97.16) | 9  (2.56) | 1  (0.28) |  |  |
|  |  | Usual activities | 257  (73.01) | 81  (23.01) | 11  (3.13) | 3  (0.85) |  |
|  |  | Pain/discomfort | 160  (45.45) | 141  (40.06) | 48  (13.64) | 3  (0.85) |  |
|  |  | Anxiety/depression | 233  (66.19) | 95  (26.99) | 23  (6.53) | 1  (0.28) |  |
| 60-69 |  | Mobility | 206  (67.76) | 76  (25.00) | 22  (7.24) |  |  |
|  |  | Self-care | 273  (89.80) | 24  (7.89) | 5  (1.64) | 2  (0.66) |  |
|  |  | Usual activities | 190  (62.50) | 84  (27.63) | 29  (9.54) | 1  (0.33) |  |
|  |  | Pain/discomfort | 120  (39.47) | 128  (42.11) | 50  (16.45) | 4  (1.32) | 2  (0.66) |
|  |  | Anxiety/depression | 172  (56.58) | 100  (32.89) | 30  (9.87) |  | 2  (0.66) |
| ≥70 |  | Mobility | 58  (29.15) | 87  (43.72) | 44  (22.11) | 5  (2.51) | 5  (2.51) |
|  |  | Self-care | 142  (71.36) | 37  (18.59) | 15  (7.54) | 2  (1.01) | 3  (1.51) |
|  |  | Usual activities | 71  (35.68) | 76  (38.19) | 39  (19.60) | 8  (4.02) | 5  (2.51) |
|  |  | Pain/discomfort | 30  (15.8) | 90  (45.23) | 70  (35.8) | 7  (3.52) | 2  (1.01) |
|  |  | Anxiety/depression | 89  (44.72) | 81  (40.70) | 26  (13.07) | 2  (1.01) | 1  (0.50) |

**Supplement 3**: Responses distributions for the EQ-5D-5L dimensions of both current study and original EQ-5D-5L valuation study

| Dimensions | Study | Level; n (%) | | | | |
| --- | --- | --- | --- | --- | --- | --- |
|  |  | 1 | 2 | 3 | 4 | 5 |
| Mobility | Current study | 1671  (83.55) | 233  (11.65) | 85  (4.25) | 6  (0.30) | 5  (0.25) |
|  | EQ-5D-5L valuation study | 873  (72.33) | 235  (19.47) | 84  (6.96) | 15  (1.24) | 0  (0) |
| Self-care | Current study | 1899  (94.95) | 73  (3.65) | 21  (1.05) | 4  (0.20) | 3  (0.15) |
|  | EQ-5D-5L valuation study | 1163  (96.35) | 32  (2.65) | 9  (0.75) | 3  (0.25) | 0  (0) |
| Usual activities | Current study | 1620  (81.00) | 276  (13.80) | 86  (4.30) | 13  (0.65) | 5  (0.25) |
|  | EQ-5D-5L valuation study | 952  (78.87) | 188  (15.58) | 60  (4.97) | 7  (0.58) | 0  (0) |
| Pain/discomfort | Current study | 1220  (61.00) | 531  (26.55) | 227  (11.35) | 17  (0.85) | 5  (0.25) |
|  | EQ-5D-5L valuation study | 571  (47.31) | 525  (43.50) | 98  (8.12) | 13  (1.08) | 0  (0) |
| Anxiety/depression | Current study | 1351  (67.55) | 479  (23.95) | 161  (8.05) | 4  (0.20) | 5  (0.25) |
|  | EQ-5D-5L valuation study | 823  (68.19) | 313  (25.93) | 62  (5.14) | 8  (0.66) | 1  (0.08) |
